# Bacteriophages as potential therapeutic agents in the control of bacterial infections

**DOI:** 10.17179/excli2025-8145

**Published:** 2025-03-31

**Authors:** Felipe Gomes Dallepiane, Malena Alejandro Coimbra Nogueira, Lucas Menezes dos Anjos, Gilberto De Souza Melo, João Paulo De Carli, Bruno Henriques, Gislaine Fongaro, Ariadne Cristiane Cabral Cruz

**Affiliations:** 1 Post-Graduation Program of Dentistry, Center for Education and Research on Dental Implants, Federal University of Santa Catarina, Florianópolis, Brazil; 2 Applied Virology Laboratory, Federal University of Santa Catarina, Florianópolis, Brazil; 3 Department of Public Health, Federal University of Santa Catarina, Florianópolis, Brazil; 4 Post-Graduation Program in Dentistry, University of Passo Fundo, Passo Fundo, Brazil; 5 Post-Graduation Program of Biotechnology and Biosciences, Federal University of Santa Catarina, Florianópolis, Brazil

## ⁯

The rapid emergence and spread of antibiotic-resistant bacteria represent a major global health issue, highlighting the urgent need to develop new antimicrobials. (Sharma et al., 2019[20]). It is estimated that, without the implementation of effective measures, antimicrobial resistance could cause up to 10 million deaths per year by 2050, surpassing the number of deaths attributed to cancer. Furthermore, the global economic impact of this crisis could reach around 100 trillion dollars, highlighting the importance of alternative treatment strategies to mitigate its devastating consequences (Piddock, 2016[16]).

Bacteriophages, also known as phages, have emerged as a promising alternative for controlling bacterial infections. It is worth noting that bacteriophages are viruses found in nature with the ability to inhibit bacterial proliferation (Richter et al., 2018[18]). Indeed, bacteriophages are the most prevalent biological entities on Earth, with an estimated 10³¹ phages dispersed across various environments (Suttle, 2005[22]). Moreover, bacteriophages are highly specific in relation to the bacteria they can infect; this specificity is a unique characteristic of phages, making them potentially valuable in therapeutic applications. They can be targeted at specific bacteria without affecting other bacteria or human cells (Elois et al., 2023[6]). 

Among the bacteriophage life cycles, two stand out as particularly important: the lytic cycle, which results in the destruction of the bacterial cell, and the lysogenic cycle, in which the phage's genetic material integrates into the bacterial genome. In Supplementary Figure 1, these two main cycles are illustrated and explained in detail. Additionally, there is a pseudolysogenic cycle, in which the phage's genetic material forms an episome. Lytic phages are often considered more suitable for therapeutic applications because their direct action on invading bacterial cells leads to a significant reduction in the bacterial population, making them a promising tool in the fight against resistant infections (Steier et al., 2019[21]). 

Bacteriophages have the ability to eliminate or control the growth of target bacteria by injecting their genetic material into host bacterial cells. This process triggers viral replication, leading to the rupture of the cell membrane and the release of new phages, which can infect other bacterial cells (Domingo-Calap and Delgado-Martínez, 2018[5]). Additionally, bacteriophages play an important role in host health and disease by preventing colonization by pathogens and degrading biofilms (Shanmugasundaram et al., 2024[19]).

In light of the growing challenge of bacterial resistance to conventional antibiotics, phages are resurfacing as one of the potential solutions to certain global public health issues (Moelling et al., 2018[14]; Ahmad et al., 2024[1]). The combined therapy of antibiotics and bacteriophages emerges as a promising alternative for treating infections. When antibiotics prove ineffective, bacteriophages can be administered alone. Additionally, the "phage-antibiotic synergy" has great potential to enhance treatment efficacy by overcoming the limitations of both methods when used separately (Bhargava et al., 2021[2]).

This growing interest in phage therapy has gained increasing attention as an innovative therapeutic option for treating bacterial infections. As a result, a significant rise in the number of scientific publications and randomized clinical trials with promising results has been observed (Leitner et al., 2017[12], 2021[13]; Gupta et al., 2019[8]; Jault et al., 2019[9]; Dobretsov et al., 2021[4]; Fedorov et al., 2023[7]; Wortelboer et al., 2023[23]; Chen et al., 2024[3]; Karn et al., 2024[10]; Kim et al., 2024[11]; Pirnay et al., 2024[17]). In addition, as of February, 2025, there are over 20 ongoing clinical trials registered in the clinicaltrials.gov database using the search terms “bacteriophage” and “bacterial infection”, of which most are sponsored by pharmaceutical industries and/or individual organizations, such as universities. The efficacy of bacteriophages, particularly against infections caused by antibiotic-resistant bacteria, has generated optimism within the scientific community, which sees them as a potentially revolutionary alternative to overcome the limitations of conventional treatments. Additionally, the ability to customize phage therapy for specific infections enhances its therapeutic potential, making it a valuable tool in medicine.

Therefore, it is crucial to conduct further clinical studies to fully explore the therapeutic potential of bacteriophages and assess their effectiveness in real-world clinical conditions. The validation of these results in humans is essential to ensure that bacteriophages can become a viable and safe solution for treating bacterial infections, especially in cases of bacterial resistance.

## Conflict of interest

None to declare

## Supplementary Material

Suppl information
