# Supplementary material for: Bacteriophages as potential therapeutic agents in the control of bacterial infections
Source: EXCLI J. 2025 Mar 31;24:524–6. doi: 10.17179/excli2025-8145 (PMC12078781; doi:10.17179/excli2025-8145)
Supplement: Suppl information [file EXCLI-24-524-s-001.pdf]

EXCLI Journal 2025;24:524-526 – ISSN 1611-2156
Received: December 30, 2024, accepted: March 18, 2025, published: March 31, 2025

Supplementary information to:

Letter to the editor:

BACTERIOPHAGES AS POTENTIAL THERAPEUTIC AGENTS IN THE CONTROL OF BACTERIAL INFECTIONS

Felipe Gomes Dallepiane*[1], Malena Alejandro Coimbra Nogueira[2], Lucas Menezes dos Anjos[1], Gilberto De Souza Melo[3], João Paulo De Carli[4], Bruno Henriques[1], Gislaine Fongaro[2,5], Ariadne Cristiane Cabral Cruz[1,2]

1 Post-Graduation Program of Dentistry, Center for Education and Research on Dental Implants, Federal University of Santa Catarina, Florianópolis, Brazil
2 Applied Virology Laboratory, Federal University of Santa Catarina, Florianópolis, Brazil
3 Department of Public Health, Federal University of Santa Catarina, Florianópolis, Brazil
4 Post-Graduation Program in Dentistry, University of Passo Fundo, Passo Fundo, Brazil
5 Post-Graduation Program of Biotechnology and Biosciences, Federal University of Santa Catarina, Florianópolis, Brazil

* **Corresponding author:** Felipe Gomes Dallepiane Post-Graduation Program of Dentistry, Center for Education and Research on Dental Implants, Federal University of Santa Catarina, 88040-900 Florianópolis, Brazil. E-mail: dallepianefe@gmail.com

https://dx.doi.org/10.17179/excli2025-8145

This is an Open Access article distributed under the terms of the Creative Commons Attribution License (https://creativecommons.org/licenses/by/4.0/).

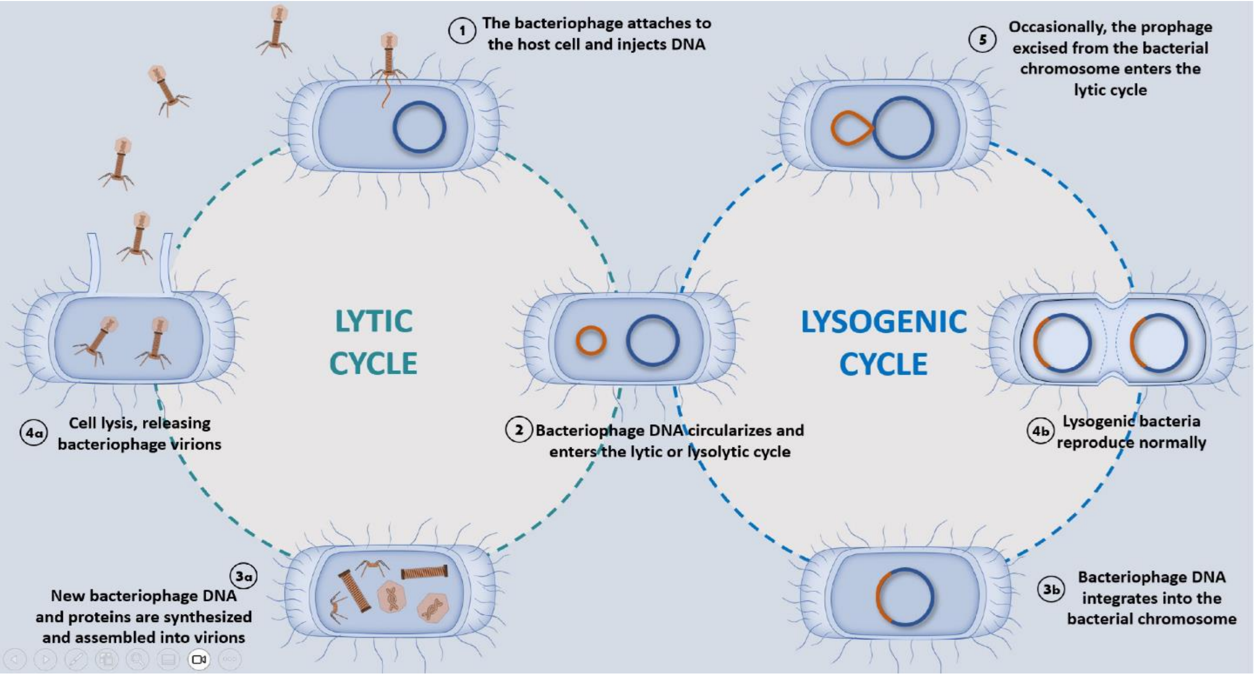

Supplementary Figure 1: Overview of the life cycles of bacteriophages: Lytic and lysogenic
